# Supplementary material for: An exploration of the use of 3D printed foot models and simulated foot lesions to supplement scalpel skill training in undergraduate podiatry students: A multiple method study
Source: PLoS One. 2021 Dec 13;16(12):e0261389. doi: 10.1371/journal.pone.0261389 (PMC8668139; doi:10.1371/journal.pone.0261389)
Supplement: S1 Appendix — (DOCX) [file pone.0261389.s001.docx]

**S1 Appendix: Interview guide for focus group**

1. Tell me about what was covered in the workshops you attended
2. What were your first impressions of the foot models?
3. How realistic did you find the ulcers?
4. How do you think the practical has prepared you for managing ulcers on an actual patient?
5. What difference do you think practising on the 3D foot models has made to how you may feel in managing ulcers on an actual patient?
6. What changes would you suggest being made to the foot models to better prepare you for patient management?
