# Supplementary material for: An exploration of the use of 3D printed foot models and simulated foot lesions to supplement scalpel skill training in undergraduate podiatry students: A multiple method study
Source: PLoS One. 2021 Dec 13;16(12):e0261389. doi: 10.1371/journal.pone.0261389 (PMC8668139; doi:10.1371/journal.pone.0261389)
Supplement: S2 Appendix — (DOCX) [file pone.0261389.s002.docx]

**S2 Appendix: Competitive State Anxiety Inventory-2 (CSAI-2) and purpose-built questionnaire (VAS)**

Name: _____________________________ Participant ID: __________________________ Date: _______________

Height: ______________________ Date of birth: / / Gender: M / F

Used a scalpel previously: Y / N

Please indicate if you have previously used a scalpel to debride callus: Y / N

Please indicate if you have observed someone use a scalpel to debride callus: Y / N

**The following questions relate to how confident you might feel just before scalpel debridement of callus in practice. Imagine you are about to use a scalpel on a client with callus and answer the following questions in relation to how you think you might feel.**

**0% would be no confidence at all whereas 100% is as confident as you’ve ever felt.**

1. How confident are you in your ability to use a scalpel?

|  | |  | |  | |  | |  | |  | |  | |  | |  | |  | |  |  |
| --- | --- | --- | --- | --- | --- | --- | --- | --- | --- | --- | --- | --- | --- | --- | --- | --- | --- | --- | --- | --- | --- |
|  | 10 | | 20 | | 30 | | 40 | | 50 | | 60 | | 70 | | 80 | | 90 | | 100 | | % |

1. How confident are you in your ability to use the appropriate grip/handhold when using a scalpel?

|  | |  | |  | |  | |  | |  | |  | |  | |  | |  | |  |  |
| --- | --- | --- | --- | --- | --- | --- | --- | --- | --- | --- | --- | --- | --- | --- | --- | --- | --- | --- | --- | --- | --- |
|  | 10 | | 20 | | 30 | | 40 | | 50 | | 60 | | 70 | | 80 | | 90 | | 100 | | % |

1. How confident are you in your ability to stabilise your hand when using a scalpel?

|  | |  | |  | |  | |  | |  | |  | |  | |  | |  | |  |  |
| --- | --- | --- | --- | --- | --- | --- | --- | --- | --- | --- | --- | --- | --- | --- | --- | --- | --- | --- | --- | --- | --- |
|  | 10 | | 20 | | 30 | | 40 | | 50 | | 60 | | 70 | | 80 | | 90 | | 100 | | % |

1. How confident are you in your ability to appropriately manipulate or stabilise the object you are debriding?

|  | |  | |  | |  | |  | |  | |  | |  | |  | |  | |  |  |
| --- | --- | --- | --- | --- | --- | --- | --- | --- | --- | --- | --- | --- | --- | --- | --- | --- | --- | --- | --- | --- | --- |
|  | 10 | | 20 | | 30 | | 40 | | 50 | | 60 | | 70 | | 80 | | 90 | | 100 | | % |

1. How confident are you in your ability to move the scalpel in the correct motion/plane?

|  | |  | |  | |  | |  | |  | |  | |  | |  | |  | |  |  |
| --- | --- | --- | --- | --- | --- | --- | --- | --- | --- | --- | --- | --- | --- | --- | --- | --- | --- | --- | --- | --- | --- |
|  | 10 | | 20 | | 30 | | 40 | | 50 | | 60 | | 70 | | 80 | | 90 | | 100 | | % |

1. How confident are you in your ability to apply the correct pressure to the scalpel?

|  | |  | |  | |  | |  | |  | |  | |  | |  | |  | |  |  |
| --- | --- | --- | --- | --- | --- | --- | --- | --- | --- | --- | --- | --- | --- | --- | --- | --- | --- | --- | --- | --- | --- |
|  | 10 | | 20 | | 30 | | 40 | | 50 | | 60 | | 70 | | 80 | | 90 | | 100 | | % |

1. How confident are you in your ability to debride a foot?

|  | |  | |  | |  | |  | |  | |  | |  | |  | |  | |  |  |
| --- | --- | --- | --- | --- | --- | --- | --- | --- | --- | --- | --- | --- | --- | --- | --- | --- | --- | --- | --- | --- | --- |
|  | 10 | | 20 | | 30 | | 40 | | 50 | | 60 | | 70 | | 80 | | 90 | | 100 | | % |

Please complete the table over the page. The questions within the table over the page relate to how confident you might feel just before scalpel debridement of callus in practice. Imagine you are about to use a scalpel on a client with callus and answer the following questions in relation to how you think you might feel.

|  | **Not at all** | **Somewhat** | **Moderately So** | **Very much So** |
| --- | --- | --- | --- | --- |
| 1. I am concerned about using a scalpel. | 1 | 2 | 3 | 4 |
| 1. I feel nervous. | 1 | 2 | 3 | 4 |
| 1. I feel at ease. | 1 | 2 | 3 | 4 |
| 1. I have self-doubts. | 1 | 2 | 3 | 4 |
| 1. I feel jittery. | 1 | 2 | 3 | 4 |
| 1. I feel comfortable. | 1 | 2 | 3 | 4 |
| 1. I am concerned I may not do as well using as scalpel as I could. | 1 | 2 | 3 | 4 |
| 1. My body feels tense. | 1 | 2 | 3 | 4 |
| 1. I feel self-confident. | 1 | 2 | 3 | 4 |
| 1. I am concerned about not performing as well as my colleagues. | 1 | 2 | 3 | 4 |
| 1. I feel tense in my stomach. | 1 | 2 | 3 | 4 |
| 1. I feel secure. | 1 | 2 | 3 | 4 |
| 1. I am concerned about choking under pressure. | 1 | 2 | 3 | 4 |
| 1. My body feels relaxed. | 1 | 2 | 3 | 4 |
| 1. I'm confident I can meet the challenge. | 1 | 2 | 3 | 4 |
| 1. I'm concerned about performing poorly. | 1 | 2 | 3 | 4 |
| 1. My heart is racing. | 1 | 2 | 3 | 4 |
| 1. I'm confident about performing well. | 1 | 2 | 3 | 4 |
| 1. I'm worried about reaching adequate proficiency with the scalpel. | 1 | 2 | 3 | 4 |
| 1. I feel my stomach sinking. | 1 | 2 | 3 | 4 |
| 1. I feel mentally relaxed. | 1 | 2 | 3 | 4 |
| 1. I'm concerned that others will be disappointed with my performance. | 1 | 2 | 3 | 4 |
| 1. My hands are clammy. | 1 | 2 | 3 | 4 |
| 1. I'm confident because I mentally picture myself reaching my goal. | 1 | 2 | 3 | 4 |
| 1. I'm concerned I won't be able to concentrate. | 1 | 2 | 3 | 4 |
| 1. My body feels tight. | 1 | 2 | 3 | 4 |
| 1. I'm confident of coming through under pressure | 1 | 2 | 3 | 4 |
