# Supplementary material for: An exploration of the use of 3D printed foot models and simulated foot lesions to supplement scalpel skill training in undergraduate podiatry students: A multiple method study
Source: PLoS One. 2021 Dec 13;16(12):e0261389. doi: 10.1371/journal.pone.0261389 (PMC8668139; doi:10.1371/journal.pone.0261389)
Supplement: S3 Appendix — (DOCX) [file pone.0261389.s003.docx]

**S3 Appendix: Raw data for quantitative studies**

S1: Participant outcomes for final year (2019) pre and post foot ulcer management workshop.

| Participant ID | T0_Cog | T0_Som | T0_SelfConf | V0_TskCon | T1_Cog | T1_Som | T1_SelfConf | V1_TskCon |
| --- | --- | --- | --- | --- | --- | --- | --- | --- |
| 1 | 12 | 14 | 25 | 81.25 |  | 12 | 29 | 80 |
| 2 | 15 | 15 | 22 | 76.25 | 17 | 15 | 27 | 80 |
| 3 | 16 | 15 | 26 | 80 |  |  |  | 87.5 |
| 4 | 18 | 16 | 26 | 88.75 | 15 | 17 | 52 | 94.375 |
| 5 | 12 | 14 | 25 | 43.75 | 10 | 11 | 24 | 80 |
| 6 | 28 | 20 | 23 | 73.75 | 19 | 14 | 27 | 92.5 |
| 7 | 13 | 14 | 22 | 70.625 | 9 | 11 | 27 | 80 |
| 8 | 16 | 11 | 26 | 56.25 | 16 | 12 | 30 | 70 |
| 9 | 10 | 12 | 28 | 78.75 | 9 | 12 | 36 | 87 |
| 10 | 18 | 14 | 23 | 31.25 | 22 | 12 | 21 | 37.5 |
| 11 | 17 | 18 | 15 | 57.5 | 12 | 15 | 21 | 67.5 |
| 12 | 31 | 27 | 15 | 42.5 | 24 | 15 | 18 | 67.5 |
| 13 | 20 | 12 | 23 | 66.25 | 12 | 11 | 22 | 77.5 |
| 14 | 16 | 14 | 17 | 68.75 | 13 | 12 | 26 | 85 |
| 15 | 22 | 20 | 19 | 70 | 20 | 12 | 22 | 80.375 |

T0 = baseline & T1 = after intervention for outcomes using the CSAI-2 =competitive state anxiety inventory-2. V0 = baseline, V1 = after intervention for outcomes using a visual analogue scale

S2: Participant outcomes for second year (2020) pre and post 1-hour training and six weeks of self-paced use of Callused 3D foot models.

| Participant ID | T0_Cog | T0_Somatic | T0_SelfConf | V0_TskCon | T1_Cog | T1_Somatic | T1_SelfConf | V1_TskCon |
| --- | --- | --- | --- | --- | --- | --- | --- | --- |
|  | 16 | 17 | 27 | 0 | 15 | 13 | 28 | 81.42857 |
|  | 21 | 19 | 28 | 7.14286 | 19 | 15 | 22 | 62.85714 |
|  | 16 | 16 | 19 | 48.5714 | 12 | 11 | 23 | 88.57143 |
|  |  |  |  | 50 |  |  |  | 82.85714 |
|  | 18 | 12 | 24 | 55.7143 | 22 | 11 | 20 | 41.42857 |
|  | 19 | 13 | 24 | 42.8571 | 12 | 14 | 30 | 85.71429 |
|  | 16 | 13 | 19 | 45.7143 | 18 | 17 | 25 | 84.28571 |
|  | 14 | 18 | 19 | 64.2857 | 21 | 15 | 22 | 65.71429 |
|  | 17 | 14 | 26 | 82.8571 | 18 | 14 | 25 | 74.28571 |
|  | 16 | 15 | 24 | 22.8571 | 18 | 20 | 27 | 67.14286 |
|  | 25 | 15 | 14 | 25.7143 | 26 | 15 | 26 | 71.42857 |
|  | 16 | 15 | 33 | 67.1429 | 15 | 12 | 35 | 70 |
|  | 27 | 26 | 13 | 20 | 20 | 14 | 23 | 47.14286 |
|  | 9 | 15 | 32 | 18.5714 | 10 | 14 | 29 | 87.14286 |
|  | 19 | 17 | 12 | 17.1429 | 19 | 11 | 23 | 62.85714 |
|  | 15 | 12 | 24 | 72.8571 | 15 | 12 | 28 | 81.42857 |
|  |  |  |  | 42.8571 |  |  |  | 61.42857 |
|  | 14 | 12 | 26 | 45.7143 | 12 | 13 | 31 | 85.71429 |
|  | 14 | 13 | 26 | 61.4286 | 18 | 15 | 26 | 81.42857 |
|  | 11 | 11 | 32 | 50 | 13 | 12 | 35 | 75.71429 |
|  | 16 | 18 | 19 | 10 | 17 | 20 | 25 | 80 |
|  | 16 | 10 | 23 | 60 | 17 | 13 | 28 | 85.71429 |
|  | 16 | 12 | 22 | 50 | 13 | 13 | 35 | 75.71429 |
|  | 16 | 22 | 22 | 44.2857 | 13 | 13 | 27 | 71.42857 |

T0 = baseline & T1 = after intervention for outcomes using the CSAI-2 =competitive state anxiety inventory-2. V0 = baseline, V1 = after intervention for outcomes using a visual analogue scale

S3: Participant data outcomes for Randomised Control Trial for second year (2019) comparisons of standard teaching vs Callused 3D foot model use.

| Participant ID | Grp | T0_Cog | T0_Som | T0_SelfCon | V0_TskCon | T1_Cog | T1_Som | T1_SelfCon | V1_TskCon |
| --- | --- | --- | --- | --- | --- | --- | --- | --- | --- |
|  | Intervention | 17 | 15 | 17 | 67.14 | 23 | 18 | 22 | 70.00 |
|  |  | 26 | 25 | 19 | 40.00 | 22 | 20 | 22 | 61.43 |
|  |  | 16 | 14 | 31 | 85.00 | 15 | 12 | 35 | 95.71 |
|  |  |  |  |  | 100.00 | 12 | 14 | 31 | 80.00 |
|  |  | 16 | 13 | 23 | 11.43 | 15 | 12 | 33 | 80.00 |
|  |  | 17 | 18 | 27 | 35.71 | 16 | 18 | 19 | 67.14 |
|  |  | 16 | 14 | 26 | 74.29 | 15 | 16 | 23 | 62.86 |
|  |  | 28 | 25 | 9 | 10.00 | 24 | 18 | 12 | 35.71 |
|  |  | 18 | 18 | 19 | 25.71 | 21 | 20 | 20 | 44.29 |
|  |  | 21 | 15 | 15 | 36.43 | 26 | 21 | 17 | 60.00 |
|  |  | 22 | 16 | 13 | 28.57 | 14 | 13 | 17 | 52.86 |
|  |  | 15 | 21 | 16 | 74.29 | 18 | 18 | 16 | 75.71 |
|  | Control | 20 | 14 | 22 | 1.43 | 18 | 18 | 18 | 60.00 |
|  |  | 12 | 14 | 24 | 57.14 | 18 | 16 | 22 | 75.71 |
|  |  |  |  |  | 31.43 | 21 | 14 | 23 | 62.86 |
|  |  | 10 | 11 | 36 | 80.00 | 13 | 17 | 31 | 84.29 |
|  |  | 18 | 15 | 25 | 18.57 | 21 | 19 | 18 | 38.57 |
|  |  | 21 | 13 | 19 | 14.29 | 19 | 14 | 20 | 42.86 |
|  |  | 21 | 20 | 18 | 48.57 | 18 | 19 | 20 | 81.43 |
|  |  | 18 | 17 | 24 | 78.57 | 17 | 18 | 23 | 58.57 |
|  |  | 10 | 12 | 33 | 80.00 | 9 | 12 | 27 | 80.00 |
|  |  | 14 | 16 | 20 | 60.00 | 23 | 24 | 22 | 81.43 |
|  |  | 16 | 13 | 21 | 35.71 | 23 | 15 | 16 | 50.00 |
|  |  | 15 | 14 | 22 | 50.00 | 10 | 14 | 30 | 81.86 |
|  |  | 18 | 17 | 16 | 50.00 | 22 | 19 | 17 | 37.14 |
|  |  | 22 | 15 | 18 | 38.57 | 18 | 16 | 12 | 54.29 |
|  |  | 21 | 17 | 20 | 50.00 | 30 | 24 | 14 | 72.86 |

T0 = baseline & T1 = after intervention for outcomes using the CSAI-2 =competitive state anxiety inventory-2. V0 = baseline, V1 = after intervention for outcomes using a visual analogue scale
